# The San Antonio Meeting

**Published:** 1903-04

**Authors:** 


### THE SAN ANTONIO MEETING.

San Antonio is making preparation to give the State Medical Association a royal welcome and to make the thirty-fifth annual meeting the most enjoyable gathering of medical men ever held in Texas. The various entertainment committees have been busily engaged for two or more months in perfecting arrangements for the proper entertainment of the four hundred or more members and visitors who are expected to attend.

The sessions will be held in Turner Hall and in the Grand Opera House, and at both places a competent bureau of information will be installed to provide guests with all needed information with reference to available rooms at the various hotels and boarding houses, rates for same, railway service, changes in the regular program, places of interest about the city, and to furnish telephone and telegraph service.

The first regular session will be called to order on the morning of April 28th, but a special preliminary meeting has been arranged for the evening of the 27th. The object of this meeting is to discuss the "reorganization question," and all members of the Association, who are interested in this important matter, are invited to be present. Dr. J. N. McCormick of Bowling Green, Ky., will attend this meeting as official representative of the American Medical Association, and will explain in detail the plan upon which the various other State Associations have been recently organized.

Transportation has been arranged with all railroads in the State so that tickets will be on sale to reach San Antonio on the 27th and 28th, and good for returning until May 2d, inclusive. Rate, one fare plus ten per cent., round trip.

Hotel Rates: Menger, $3.00 per day; the Mahncke, $2.50 per day; Maverick, $2.00 to $2.50 per day (European plan $1.00); the Bexar, $2.50 per day; Alamo Flats, $1.50 per day; Southern, $2.50 per day; Elite, $1.00 per day (European plan).

The management of the Hot Sulphur Wells will offer free use of the hot sulphur baths during the entire meeting to all visiting physicians.

During the entire five days meeting the recently completed fire-proof Physicians and Surgeons Hospital will be thrown open for the inspection of all visitors. This institution, planned and built entirely by physicians, and to be run by physicians in the interest of the medical profession, is the best of the kind in the South, and is well worth a visit. It will not be opened to receive patients until after the adjournment of the State Association.

This meeting should not only be the most important, but the best attended and most interesting meeting ever held in the State.

On the morning of the 28th, the session will be opened with

Invocation ..............................Rev. Arthur Jones
Address of Welcome, Citizens.............Hon. William Aubrey
Address of Welcome, Administration...........Hon. J. E. Webb
Address of Welcome, Local Profession............Dr. F. Paschal
Response by the President of the Association........Dr. S. E. Red

On the evening of 28th, there will be an "old fashion" barbecue at the famous Hot Wells Hotel, followed by a reception and dance.

On the evening of 30th, there will be a reception and ball at Turner Hall. These "social functions" will be under the management of the Ladies' Committee, and they will be brilliant and enjoyable. San Antonio always does the right thing in the swellest and most graceful manner.

To date of going to press the program of the meeting has not been received. Secretary West will mail a copy to each member before this is in the hands of readers. Several distinguished visitors will be present, and "a feast of papers and a flow of 'Bud' " is in store. This will be an epoch marking meeting, a Red Letter Day, in the history of the Association.

